# Determining factors associated with breastfeeding and complementary feeding practices in rural Southern Benin

**DOI:** 10.1002/fsn3.1971

**Published:** 2020-11-06

**Authors:** Fifali Sam Ulrich Bodjrènou, Waliou Amoussa Hounkpatin, Céline Termote, Geoffroy Dato, Mathilde Savy

**Affiliations:** ^1^ Alliance of Bioversity International and CIAT Cotonou Benin; ^2^ University of Abomey‐Calavi, Faculty of Agricultural Sciences Abomey‐Calavi Benin; ^3^ Alliance of Bioversity International and CIAT Nairobi Kenya; ^4^ NUTRIPASS‐IRD Cotonou Benin

**Keywords:** Benin, Breastfeeding practices, Complementary feeding practices, Determinants

## Abstract

This study aimed at characterizing breastfeeding and complementary feeding practices in a food‐insecure area of Benin and identifying factors associated with these practices. A cross‐sectional study was conducted in the districts of *Bopa* and *Houéyogbé* among *n* = 360 mother–child pairs. Children aged 0–17 months were considered. Socioeconomic characteristics among children and mothers, *Breastfeeding on demand*, *Breastfeeding frequency during children illness,* and *Positioning and Attachment of children while breastfeeding* were assessed using semi‐structured interviews and observations. Qualitative 24‐hr recalls were administered to mothers to compute WHO recommended complementary feeding practices indicators namely *minimum dietary diversity* (MDD), *minimum meal frequency* (MMF), and *minimum acceptable diet* (MAD) among 6–17 months old children (*n* = 232). Associations between each feeding practice and mothers' socioeconomic characteristics were tested using multivariate generalized linear models. *Breastfeeding on demand* and *good positioning and attachment for breastfeeding* rates were 59% and 66%, respectively. Only 26% of mothers used to increase breastfeeding frequency when their children were ill. The proportions of children who met MDD, MMF, and MAD were 51%, 75%, and 44%, respectively. Children living in *Houéyogbé* were less likely to be breastfed on demand compared with those living in *Bopa*; however, they had better *breastfeeding frequency during illness* and meal frequency. Socioeconomic factors with significant association with breastfeeding practices were children age and sex and mothers’ education, ethnicity, and employment status. Complementary feeding practices were positively associated with children's age but not with other socioeconomic characteristics. Breastfeeding and complementary feeding practices were almost suboptimal or medium and still need to be improved through well designed nutrition intervention program including nutrition education.

## Graphical abstract

In the present study, we evaluated breastfeeding and complementary feeding practices among children aged, respectively, 0–17 months and 6–17 months in a rural and food‐insecure area in Southern‐Benin, West Africa. We determined also socioeconomic and agricultural factors, which were associated to these practices in order to well design a nutrition education program. This graph presents the percentages of children who have achieved recommended breastfeeding and complementary feeding practices in the rural districts of Bopa and Houeyogbe (Southern Benin).

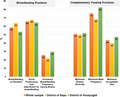

## 1 INTRODUCTION

Benin is a West African country where child malnutrition is a public health problem. According to the latest Demographic and Health Survey (DHS), about one‐third of children under 5 years old were stunted in 2017‐2018 (INSAE & ICF, 2019). A major contributor to this situation is the inadequacy of breastfeeding and complementary feeding practices. About one‐half of newborns (54%) benefit from early initiation of breastfeeding (INSAE & ICF, 2019). The median duration of exclusive breastfeeding (EBF) was 2.4 months in rural areas and 1.3 months in urban areas, and the rate of EBF till six months of age barely reached 42% (INSAE & ICF, 2019). The diversity of complementary foods was low with only 28% of 6–23 months children who met the minimum dietary diversity (MDD) (INSAE & ICF, 2019).

A diagnostic study conducted in two districts of southern Benin (namely *Bopa* and *Houéyogbé*) located in a high food‐insecure area reported that infants and young children feeding practices were suboptimal (Mitchodigni, et al., 2017a; Mitchodigni, et al., 2017b). Authors suggested that multisectoral interventions should be implemented in this area to improve feeding practices for young children. Following this recommendation, a nutrition education program was planned in this region aiming at optimizing mothers’ knowledge on nutrition and subsequently, breastfeeding and complementary feeding practices. Nutrition education is meant to improve knowledge, skills, motivation, and behavior of individuals or communities, leading potentially to subsequent positive effects on nutritional status and health (FAO, 2005). In sub‐Saharan Africa, nutrition education interventions showed good contribution to improving breastfeeding practices (Aidam et al., 2005; Tylleskär et al., 2011), complementary feeding practices (Waswa et al., 2015) and also nutritional status of <2 years children (Lassi et al., 2013).

In order to refine the messages' content and take into account the contextual factors as part of the intervention, an in‐depth understanding of the situation toward breastfeeding, complementary feeding practices, and associated factors in the area was necessary. Evidence from Benin and other African countries shows that multiple factors have the potential to affect positively or negatively breastfeeding and feeding practices among young children. These factors include, but are not limited to, cultural beliefs and habits (Amoussa Hounkpatin et al., 2014; Aborigo et al., 2012; Aryeetey & Goh, 2013; Issaka et al., 2014; Otoo et al., 2009), households' socioeconomic status (Agho et al., 2011; Mitchodigni, et al., 2017b; Sokan‐Adeaga et al., 2019), agriculture practices within the households (Mitchodigni, et al., 2017b), mothers' overall instruction (Olatona et al., 2017; Qureshi et al., 2011; Sokan‐Adeaga et al., 2019), mothers' occupation (Amoussa Hounkpatin et al, 2014; Mitchodigni, et al., 2017b; Nkrumah, 2017), as well as knowledge and perceptions of appropriate practices (Aborigo et al., 2012; Agunbiade & Ogunleye, 2012; Aidam et al., 2005; Issaka et al., 2014; Otoo et al., 2009; Qureshi et al., 2011).

The present study, which was conducted before the implementation of the nutrition education program, aimed at (a) characterizing breastfeeding and complementary feeding practices in the intervention area using indicators which addressed directly the limitations among children feeding practices identified during the diagnostic survey and (b) identifying factors that were associated with these practices specifically in this intervention area. This could help to identify factors other than those relative to the intervention which could also influence the effect of the intervention by affecting the ability of the beneficiaries to adopt or reject recommended practices.

## 2 MATERIALS AND METHODS

### 2.1 Setting

The study was conducted in the districts of *Bopa* and *Houéyogbé* which present the highest rates of household food insecurity in the Mono department, respectively 40% and 34% (INSAE & PAM, 2014).

### 2.2 Sampling

The data presented in this paper were related to the baseline survey of the impact evaluation of the nutrition education intervention implemented in the districts of Bopa and Houéyogbé in Southern Benin (Bodjrenou et al., 2020). A total of eight villages were randomly selected after stratification by district. In each village, 45 mother–child pairs were randomly selected from an exhaustive list of 0 to 17‐months‐old children living in the villages using a random number technique. We targeted this age‐group firstly to focus on breastfeeding and complementary feeding practices. Secondly, the intervention and its impact evaluation were meant to last for 6 months; hence, children would be 6–23 months old at endline. In total, 360 mother–child pairs were surveyed.

### 2.3 Data collection

Mothers or primary caregivers of children were interviewed during home visits by trained enumerators. Mothers' socioeconomic characteristics including age, ethnic group, employment status, marital status, level of education, and participation in nutrition education programs were collected. *Breastfeeding on demand* and attitudes toward *breastfeeding frequency during child illness* were recorded, while correct *positioning and attachment of the child during breastfeeding* was observed. We considered six parameters during the observations: breasts held by mother's hand, mother's hand doing a "C‐shape," child's chin attached to the breast, child's lip in eversion, child in straight position, and child's whole body facing the mother's chest (Unicef, 2012; Vinther & Helsing, 1997; WHO & UNICEF, 1993). Enumerators observed mothers breastfeeding their children; each correct *breastfeeding positioning and attachment* parameter adopted by mothers was marked “1,” and “0” otherwise.

The enumerators stayed in the study villages for about 2 weeks. This gave them several opportunities to observe mothers' practices. If an enumerator did not have the opportunity to observe a breastfeeding episode when interviewing a mother, she/he did not ask the mother to breastfeed her child, as this might disturb the habits. Enumerators had taken advantage of their stay in the village and would come back to make the observation as soon as he would have the opportunity to see the child at the breast. There is no specific duration of a breastfeeding episode. We have observed that youngest children breastfed longer and more frequently than older ones. Enumerators recorded different parameters of breastfeeding positioning and attachment as soon as mother positioned child and started breastfeeding.

The children aged 6–17 months feeding practices were also assessed using a qualitative 24 hr recall (WHO et al., 2008). All foods and drinks that had been used to feed the child the previous day and their constitutive ingredients were listed by the mother or the primary caregiver.

### 2.4 Data management and statistical analysis

The following binary variables were computed and analysed: (a) *Breastfeeding on demand* (yes/no) (b) *Breastfeeding frequency when the children were ill* (increased versus unchanged or decreased). For each mother, a *breastfeeding positioning and attachment* score was computed by summing up the mark attributed to each of the six positioning parameters that were considered during the observations. The score, which theoretically ranged from 0 to 6 points, was recoded as a binary variable using the median score as a threshold: (a) mothers with a score less than the median and (b) mothers with a score equal or higher than the median (hence considered as “*good positioning*”).

From the 24 hr recall data, foods were categorized into seven food groups as recommended namely: (a) grains, roots, and tubers; (b) legumes and nuts; (c) dairy products; (d) flesh foods; (e) eggs; (f) vitamin‐A rich products; (g) fruits and vegetables different from those rich in vitamin‐A (Kennedy et al., 2010; WHO et al., 2008). The minimum dietary diversity (MDD), minimum meal frequency (MMF), and minimum acceptable diet (MAD) were computed following WHO and UNICEF guidelines (Kennedy et al., 2010; WHO et al., 2008). Children aged 6–23 months were considered having met MDD if they had consumed foods from at least four different food groups out of the seven recommended over the day prior to the survey. Children who received solid, semi‐solid, or soft foods (including milk feeds for nonbreastfed children) the minimum number of times or more the day prior to the survey were considered having met MMF. A child who met both MDD and MMF was considered having met MAD (WHO et al., 2008). We, then, determined the proportions of children having met MDD, MMF, and MAD.

Characteristics of mothers and feeding practices indicators were described and presented using descriptive statistics: percentages for categorical variables and mean ± *SD* for continuous variables. Comparisons among the two districts were also performed using chi‐square tests for categorical variables and Mann–Whitney test or Student's *t* tests for continuous variables. Associations between socioeconomic characteristics of mothers and breastfeeding and feeding practices outcomes were analysed through a two‐step approach. Bivariate analyses (chi‐square, Mann–Whitney, and Student's *t* tests) were first performed to identify factors with significant association with each practice. Variables that were significant at 20% (Mickey & Greenland, 1989; Trekpa et al., 2005) were kept for the next step. Secondly, we used generalized linear model (GLM) with binomial distribution probability and logit link function to build models presenting the contribution of the variables (those that were significant in bivariate analysis) to each outcome. All independent variables were entered together into each model in one step. Data presented included odd‐ratios, confidence interval associated, z‐value, and *p*‐value.

The relevance of regression models was assessed by displaying the goodness of fit parameters (X‐squared and *p*‐value) associated with Hosmer–Lemeshow test (*p*‐value should be higher than 5%) and the error rate (quality of model's predictions) derived from the confusion matrix (error rate should be <0.5). Where Hosmer–Lemeshow test was not significant, we performed Pearson's residuals test (*p*‐value should be <5%).

Descriptive analyses and bivariate analyses were carried out using SPSS 23.0 (IBM‐SPSS, 2014) while GLM and relative tests were conducted using R (Zuur et al., 2013). All analyses took into account the study design.

## 3 RESULTS

### 3.1 Socioeconomic characteristics of the sample

The average ages were 8.4 ± 4.9 months for children and 27.5 ± 5.9 years for mothers. Boys represented 53.1% of children in the sample. Most of mothers were from the *Sahouè* ethnic group, had no schooling, and lived with their husbands (Table 1). Children's characteristics (gender and age) were similar across districts. Mothers' age, marital status, and ethnic group were also similar across districts. However, the proportion of mothers with no schooling was significantly lower in the district of *Houéyogbé* than *Bopa*, with 45.0% and 77.2% respectively (*p*‐value <.001).

Only 13.9% of mothers had no income generating activity. Mothers living in Bopa were mainly involved in agriculture (48.3%), trading (39.4%), and food processing (26.7%) while those living in Houéyogbé were involved in agriculture (27.2%), trading (23.9%), foods selling (24.4%), food processing (23.3%), and handicraft (20.6%) (Table 1).

Less than 6% of mothers had benefitted from a nutrition education program in the past with a difference between districts (Bopa: 2.2%; Houéyogbé: 9.4%; p‐value =.003) (Table 1).

**TABLE 1:** Socioeconomic characteristics of children and mothers (*n* = 360)

Parameters	n		All	Bopa	Houéyogbé	p‐value
**Children characteristics**						
Age (months)	360		8.4 (4.9)	8.5 (4.8)	8.2 (5.0)	.578^*S*^
Gender						
Male	191		53.1	50.6	55.6	.342^*C*^
Female	169		46.9	49.4	44.4	
**Mothers characteristics**						
Age (years)	358*		27.5 (5.9)	28.1 (5.9)	26.9 (5.8)	.054^*S*^
Marital status						
Living alone	73		20.3	16.7	23.9	.088^*C*^
Living with husband	287		79.7	83.3	76.1	
Formal education level						
No schooling	220		61.1	77.2	45.0	<.001^*C*^
Literate or primary school	95		26.4	21.1	31.7	
Secondary school and more	45		12.5	1.7	23.3	
Ethnic group						
Sahoué	295		81.9	82.2	81.7	.891^*C*^
Other	65		18.1	17.8	18.3	
Employment status						
No activity	50		13.9	11.1	16.7	.128^*C*^
Food processing	90		25.0	26.7	23.3	.465*^C^*
Agriculture	136		37.8	48.3	27.2	<.001^*C*^
Animal breeding	29		8.1	7.8	8.3	.846^*C*^
Trading	114		31.7	39.4	23.9	.002^*C*^
Foods selling	64		17.8	11.1	24.4	.001^*C*^
Handicraft	45		12.5	4.4	20.6	<.001^*C*^
Number of activities			1.4 (1.0)	1.5 (0.9)	1.3 (1.0)	.178^*M*^
Attended nutrition education programs in the past						
At least once		21	5.8	2.2	9.4	.003^*C*^
Never		339	94.2	97.8	90.6	

Note

Values presented are percentage for categorical variables and Mean (Standard Deviation) for continuous variables. *p*‐values presented are probabilities relative to chi‐square test (^C^) for categorical variables, Student's *t* test for Ages (^S^) and Mann–Whitney test (^M^) for Number of activities.

* Two mothers did not know and could not estimate their exact age; therefore, we missed this information.

### 3.2 Feeding practices

**FIGURE 1:**
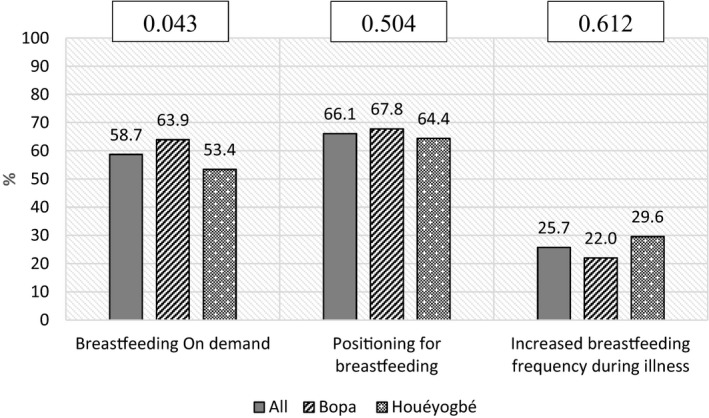
Breastfeeding practices in the sample and by district: Percentage of children achieving *Breastfeeding on Demand*, *Good Positioning and attachment for breastfeeding* and Increased *Breastfeeding Frequency during illness. (% = Percentage; p‐values presented are probabilities relative to chi‐square test comparing the two districts)*

All children in our sample were breastfed. Percentage of mothers who used to breastfeed on demand their children were 58.7% (Figure 1); this proportion was slightly higher in *Bopa* than *Houéyogbé* (63.9% and 53.4%, respectively; *p*‐value = 0.043). The proportion of mothers who had met *good positioning and attachment for breastfeeding* was 66.1% with no significant difference across districts (Figure 1). Only one‐quarter of mothers declared increasing *breastfeeding frequency when their children were ill*.

**FIGURE 2:**
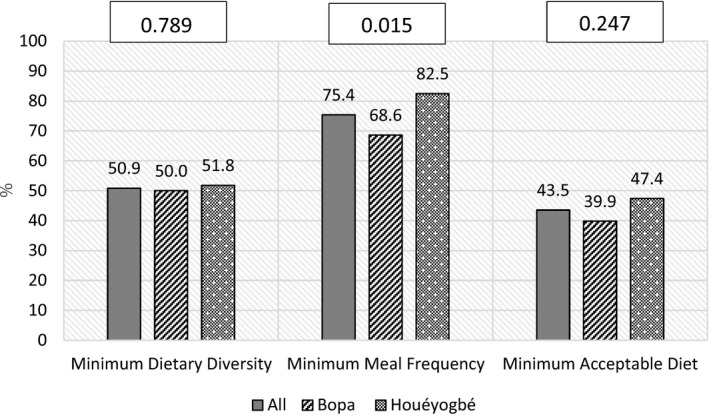
*Complementary feeding practices in the whole sample and by district: Percentage of children achieving recommended complementary feeding practices. (% = Percentage; p‐values presented are probabilities relative to chi‐square test comparing the two districts)*

The percentages of children meeting the requirements of MDD, MMF, and MAD were respectively 50.9%, 75.4%, and 43.5% (Figure 2). In Houéyogbé, 82.5% of children met the MMF while they were only 68.6% in Bopa (*p*‐value =.015). The other indicators did not differ across districts.

### 3.3 Factors associated with breastfeeding practices

Mothers who lived in *Houéyogbé* were less likely to breastfeed on demand (OR = 0.59, CI 95% = [0.36; 0.98], *p* = .04) than mothers who lived in the district of *Bopa* (Table 2). Mothers who had higher school level (at least secondary school) were 3.08 more likely to breastfeed on demand than mothers with no schooling (OR = 3.08, CI 95% = [1.40; 6.79], *p* = .005). Likewise, mothers who practiced trading and those who were not from the *Sahouè* ethnic group had higher odds to breastfeed on demand than others (Table 2).

Regarding good positioning and attachment for breastfeeding, only the children's age was associated with this indicator. Mothers of older children tended to have lower odds of having good positioning and attachment for breastfeeding compared with others (OR = 0.94, CI 95% = [0.90; 0.99], p = .01) (Table 2).

Several factors were associated with the frequency of breastfeeding during illness episodes. Increased breastfeeding was more likely to happen among mothers who lived in Houéyogbé compared with those living in Bopa (OR = 2.16, CI 95% = [1.12; 4.18], p = .02), when children were older (OR = 1.10, CI 95% = [1.02; 1.19], p = .02), and when they were boys (OR = 0.51, CI 95% = [0.27; 0.96], p = .04). Unexpectedly higher the education level of mothers was, lesser was the likelihood to increase the frequency of breastfeeding illness episodes (OR = 0.19, CI 95% = [0.08; 0.48], p = .0004) (Table 2).

**TABLE 2:** Results from the GLM on breastfeeding practices

Parameters	OR	Confidence interval associated to OR		Z‐value	*p*‐value
		2.50%	97.50%		
Breastfeeding on demand (*n* = 356; Hosmer and Lemeshow goodness of fit: X‐squared = 11.03, *df* = 8, *p*‐value = .1999; Error rate = 38.48)					
(Intercept)	0.62	0.33	1.19	−1.42	.15471
District_*Houéyogbé (Ref = Bopa)*	0.59	0.36	0.98	−2.04	.04104
Gender_*Female (Ref = Male)*	0.96	0.61	1.50	−0.19	.84630
Education_*Literate or Primary school (Ref = No schooling)*	1.44	0.84	2.45	1.34	.18083
Education_*Secondary school (Ref = No schooling)*	3.08	1.40	6.79	2.79	.00526
Ethnic group_*Others (Ref = Sahouè)*	1.93	1.04	3.57	2.10	.03583
Agriculture_*Yes (Ref = No)*	1.72	0.90	3.28	1.63	.10271
Trading_*Yes (Ref = No)*	1.89	1.07	3.35	2.20	.02817
Children's age (months)	1.05	1.00	1.10	1.91	.05591
Number of activities	1.00	0.70	1.42	−0.01	.99260
Good positioning and attachment for breastfeeding (*n* = 358; Hosmer and Lemeshow goodness of fit: X‐squared = 4.81, *df* = 8, *p*‐value = .7775; Error rate = 32.68)					
(Intercept)	3.48	1.77	6.84	3.62	.00030
District_*Houéyogbé (Ref = Bopa)*	0.87	0.55	1.39	−0.57	.56751
Gender_*Female (Ref = Male)*	1.20	0.76	1.90	0.77	.44025
Ethnic group_*Others (Ref = Sahouè)*	1.53	0.82	2.85	1.33	.18406
Animal breeding_*Yes (Ref = No)*	0.47	0.18	1.23	−1.54	.12467
Trading_*Yes (Ref = No)*	0.61	0.36	1.05	−1.79	.07306
Children age	0.94	0.90	0.99	−2.47	.01369
Number of activities	1.14	0.84	1.55	0.86	.38758
Food selling_*Yes (Ref = No)*	0.54	0.29	1.00	−1.97	.04859
Increasing breastfeeding frequency during illness (*n* = 256; Hosmer and Lemeshow goodness of fit: X‐squared = 9.68, *df* = 8, *p*‐value = .2881; Error rate = 26.17)					
(Intercept)	0.07	0.01	0.48	−2.71	.00674
District_*Houéyogbé (Ref = Bopa)*	2.16	1.12	4.18	2.29	.02179
Gender_*Female (Ref = Male)*	0.51	0.27	0.96	−2.10	.03585
Marital status_*Living With husband (Ref = Living Alone)*	1.88	0.78	4.54	1.40	.16268
Education_*Literate or Primary school (Ref = No schooling)*	0.19	0.08	0.48	−3.53	.00042
Education_*Secondary school (Ref = No schooling)*	0.54	0.19	1.57	−1.13	.25849
Food processing_*Yes (Ref = No)*	1.38	0.70	2.74	0.92	.35523
Animal breeding_*Yes (Ref = No)*	1.32	0.49	3.51	0.55	.58170
Children age	1.10	1.02	1.19	2.33	.01985
Mother age	1.01	0.95	1.07	0.29	.76961

Abbreviations: OR, odd‐ratio; Ref, modality of reference.

### 3.4 Factors associated with complementary feeding practices

Very few factors were associated with the three indicators on complementary feeding practices (Table 3). Only the age of children was positively associated with the MDD (OR = 1.13, CI 95% = [1.04; 1.23], *p* = .006) and the MAD (OR = 1.09, CI 95% = [1.01; 1.19], *p* = .04). Children who lived in *Houéyogbé* were more likely to meet the MMF compared to children who lived in *Bopa* (OR = 2.47, CI 95% = [1.24; 4.93], *p* = .01). However, the district was not associated with the MMD or the MAD. The variables related to the mothers' education and employment were not associated with any of the complementary feeding practices indicators.

**TABLE 3:** Results from the GLM on complementary feeding practices

Parameters	OR	Confidence interval associated to OR		Z‐value	*p*‐value
		2.50%	97.50%		
Minimum dietary diversity MDD (*n* = 230; Hosmer and Lemeshow goodness of fit: X‐squared = 5.91, *df* = 8, *p*‐value = .6573; Error rate 41.30)					
(Intercept)	0.37	0.12	1.14	−1.73	.08421
District_*Houéyogbé (Ref = Bopa)*	0.95	0.55	1.64	−0.17	.86438
Gender_*Female (Ref = Male)*	1.04	0.60	1.79	0.14	.89179
Nutrition education_*Yes (Ref = No)*	2.37	0.67	8.42	1.34	.18186
Food processing_*Yes (Ref = No)*	0.69	0.35	1.36	−1.06	.29011
Children age (months)	1.13	1.04	1.23	2.75	.00589
Number activities	0.82	0.59	1.14	−1.17	.24133
Minimum meal frequency MMF (*n* = 230; Hosmer and Lemeshow goodness of fit: X‐squared = 10.76, *df* = 8, *p*‐value = .2157; Error rate = 24.35					
(Intercept)	1.64	0.51	5.32	0.83	.40960
District_*Houéyogbé (Ref = Bopa)*	2.47	1.24	4.93	2.57	.01020
Gender_*Female (Ref = Male)*	1.33	0.71	2.50	0.90	.37000
Education_*Literate or Primary school (Ref = No schooling)*	1.34	0.60	2.99	0.72	.47360
Education_*Secondary school (Ref = No schooling)*	0.63	0.22	1.78	−0.88	.38110
Children age	1.01	0.92	1.11	0.20	.84050
Minimum acceptable diet MAD (*n* = 230; Hosmer and Lemeshow goodness of fit: X‐squared = 31.33, *df* = 8. *p*‐value = .0001229; Pearson's Residuals: 0.3878219; Error rate = 38.26)					
(Intercept)	0.31	0.10	0.97	−2.01	.04430
District_*Houéyogbé (Ref = Bopa)*	1.20	0.69	2.09	0.64	.52060
Gender_*Female (Ref = Male)*	1.22	0.71	2.10	0.71	.47850
Food processing_*Yes (Ref = No)*	0.79	0.39	1.60	−0.65	.51370
Children age	1.09	1.01	1.19	2.07	.03810
Number of activities	0.79	0.56	1.11	−1.38	.16860
Food selling_*Yes (Ref = No)*	1.88	0.92	3.85	1.73	.08300

Abbreviations: OR, odd‐ratio; Ref, modality of reference.

## 4 DISCUSSION

Results from our study showed that children feeding practices were suboptimal or medium and this situation requires actions for improvement. The definition of adequate interventions requires first to have a good knowledge of the situation and associated factors. Thus, we observed that age and sex of children, district of residence, ethnic group, education level, and employment status of mothers were associated with breastfeeding practices, with some variations depending on the indicators used. Mothers' education was positively associated with *breastfeeding on demand* but was negatively associated with *breastfeeding frequency during illness*. Educated mothers are likely to have a better knowledge of the importance of good breastfeeding practices (Al Ketbi et al., 2018), and we would expect better practices among them. On the other hand, educated mothers are also more likely to be employed in public or private companies and they may have less time to apply good practices compared to mothers with informal jobs or no job at all. Employed mothers may also be stressed and tired, resulting in little time or energy at the end of the day to nurse their babies properly (Netshandama, 2002). Moreover, home‐based activities like agriculture or small trading offer to mothers the opportunity to stay at home or nearby. Other activities such as food selling require to leave home, and this may affect time spent with children and breastfeeding practices (Amoussa Hounkpatin et al, 2014; Nkrumah, 2017). Mother's marital status was not associated with breastfeeding practices in our study. Yet, a study in Ghana revealed better breastfeeding practices among women who lived with their husband (Rose, 2007). Indeed, when husbands have good financial situation, women could benefit from these resources and devote more time to nursing roles and less to income generating activities.

We also found that mothers living in Bopa, who were mostly involved in home‐based activities, were more likely to breastfeed on demand than those living in Houéyogbé. The fact that Houéyogbé is more urbanized than Bopa may explain these results (Mitchodigni, et al., 2017b). These results are in line with national DHS (INSAE & ICF, 2019; INSAE, 2015) and other studies from low‐ and middle‐income countries (Hitachi et al., 2019; Iffa & Serbesa, 2018; Kumar et al., 2017) which reported that most of breastfeeding practices, especially *breastfeeding on demand* or EBF, were better in rural than urban areas. On the other hand, level of urbanization could be positively associated with other practices. For example, when a child is sick, Beninese mothers living in rural areas would preferentially treat their children with medicinal herbs in the form of tisanes (Allabi et al., 2011; Towns et al., 2014); the intakes of these tisanes could reduce breastmilk consumption. This echoed with our finding: mothers living in Houéyogbé were more likely to increase *breastfeeding frequency when children were ill* than mothers living in Bopa.

*Breastfeeding on demand* varied according to mothers’ ethnic groups. Children from the *Sahouè* ethnic group were less likely to benefit from *breastfeeding on demand* than others. As shown in other sub‐Saharan contexts (Asare et al., 2018; Jacdonmi et al., 2016; Tawiah‐Agyemang et al., 2008; Wanjohi et al., 2017), social and cultural beliefs were determinant for breastfeeding practices. Finally, children's age was negatively associated with the likelihood to adhere to the recommended positions for breastfeeding. Mothers explained during informal discussions that as children were growing up, they became stronger and more agitated; consequently, it was more difficult to control them during breastfeeding.

Regarding complementary feeding, as expected, we observed that children's age was positively associated with the MDD and MAD. Generally, mothers used to replace breast milk with porridge and then family foods progressively as children are growing up. Traditional porridges that are first given to young children consist of a cereal mixed with water and sometimes sugar, which provide low dietary diversity. The family meals that are eaten by older children offer higher dietary diversity. Thus, as children are growing up and start eating family meals, their dietary diversity increases (Amoussa Hounkpatin et al, 2014).

Living in Houéyogbé also seemed to be more favorable to higher meal frequency among children than living in Bopa; probably because of the difference of urbanization (Mitchodigni, et al., 2017b). No other factors were found to be associated with the complementary feeding indicators we investigated.

We observed that some intercepts were significant (*Increasing* *Breastfeeding Frequency during Illness*, *Good Positioning, and Attachment for Breastfeeding*, minimum acceptable diet). Thus, there were possibly other factors influencing feeding practices, but these factors were not collected in the present study.

Mitchodigni, et al. (2017b) showed also that agriculture, especially the diversity of food groups grown by households, increased the likelihood of meeting the MDD among 6–23 months old children. Moreover, we expected that participating in a nutrition education program in the past would have been associated with present feeding practices. Indeed, many studies had shown that nutrition education interventions targeting breastfeeding or dietary habits had good contribution to improving breastfeeding and complementary feeding practices in low‐ and middle‐income countries especially in Africa (Aidam et al., 2005; Lassi et al., 2013; Tylleskär et al., 2011; Waswa et al., 2015). In other cases, nutrition education helped to increase knowledge and attitudes but not practices (Mojisola et al., 2019; Ruzita et al., 2007). However, in the present study few mothers (<6%) participated this program and this could not allow good analysis or conclusions. Moreover, participating a nutrition program does not necessary lead to behavior change and achieving the adequate knowledge does not guarantee the adoption of good practices. Some other factors such as limited access to nutritious foods (Dang et al., 2005), financial constraints (Aborigo et al., 2012; Otoo et al., 2009), and pressures or support from families including food taboos (Kakute et al., 2005; Amoussa Hounkpatin et al, 2014; Issaka et al., 2015) play an important role. In the other hand, nutrition education programs have to be well planned and delivered (Chapman‐Novakofski, 2014) and intensive (Bukusuba, 2005; Roy et al., 2005) to ensure their efficiency .

### 4.1 Limitation

We could have used qualitative method in the present study to have in‐depth understanding of the situation. Qualitative research approaches allow a detailed description of participants' feelings, opinions, experiences, and interpretations of their actions (Rahman, 2017). However, they have some limitations. Results cannot be generalized to the entire research population with the same degree of certainty as quantitative measures. Sample size used in qualitative research is often smaller than that used in quantitative research methods (Dworkin, 2012). Qualitative research results are not tested to determine whether they are statistically significant or due to chance (Atieno, 2009). Therefore, we decided to use quantitative method to ensure measurability and comparability (statistical) of indicators since we aim to collect data related to the same indicators at the end of the intervention.

## 5 CONCLUSION

Breastfeeding and complementary feeding practices were not optimal in the districts of Bopa and Houéyogbé, department of Mono, Southern Benin. These results supported the importance of a nutrition education program at the community level in order to improve mothers’ and community members’ knowledge and attitudes toward children feeding practices. Moreover, socioeconomic, cultural, and demographic factors such as age and sex of children, district of residence, ethnic group, education level, and employment status identified in the present paper as influencing infants and children feeding practices will be taken into account when designing the community‐based nutrition education program. According to these factors, different categories of people within our target population will be defined. Thus, key messages will be adapted to address each of these categories considering their specific backgrounds in terms of knowledge, occupation, food habits, culture, and beliefs.

## CONFLICT OF INTEREST

"No conflicts of interest."

## ETHICAL APPROVAL

The study was conducted in accordance with the Declaration of Helsinki. Ethical clearance was obtained from the Benin National Ethics Committee for Scientific Research (N°45/MS/DC/SGM/DFR/CNERS/SA). Administrative authorities of the two districts were informed and approved the study. Written informed consent of participants was obtained after receiving complete information about the study in the local language.
